# Dual-Stage Crosslinking of Gelatin-Alginate Bioink Supplemented with Wharton’s Jelly to Generate 3D Bioprinted Scaffolds for Wound Healing Application

**DOI:** 10.3390/polym18111331

**Published:** 2026-05-28

**Authors:** Nghia Thi Hieu Phan, Nho Thuan Nguyen, Ha Le Bao Tran, My Thi Ngoc Nguyen

**Affiliations:** 1 Laboratory of Tissue Engineering and Biomedical Materials, University of Science, Ho Chi Minh City 700000, Vietnam; phtnghia@hcmus.edu.vn (N.T.H.P.); ngtnho@hcmus.edu.vn (N.T.N.); tlbha@hcmus.edu.vn (H.L.B.T.); 2 Department of Physiology and Animal Biotechnology, Faculty of Biology and Biotechnology, University of Science, Ho Chi Minh City 700000, Vietnam; 3 Vietnam National University, Ho Chi Minh City 700000, Vietnam

**Keywords:** bioprinting, extracellular matrix, Wharton’s jelly, tissue engineering, wound healing, gelatin-alginate, crosslinking

## Abstract

Incorporation of extracellular matrix (ECM) components into bioinks can enhance biological functionality but often compromises print fidelity and structural stability. This study developed a dual-stage calcium chloride (CaCl_2_) crosslinking strategy to incorporate Wharton’s jelly-derived ECM (WJ-ECM) into a gelatin-alginate bioink for bioprinted scaffold fabrication. A baseline formulation (BGA) and a WJ-ECM-supplemented formulation (BGAE, 1 mg/mL) were pre-crosslinked with 14 mM CaCl_2_ prior to extrusion, followed by secondary crosslinking in 0.5 M CaCl_2_ post-printing. Both formulations exhibited comparable viscosity (20–180 kcP) and high print fidelity (Pr = 0.99 ± 0.01 for BGA; 0.95 ± 0.01 for BGAE), with scaffolds displaying well-defined architecture and over 84% of pores within the target range (160–270 µm). FTIR analysis confirmed WJ-ECM incorporation without detectable alteration of the primary matrix structure. Both scaffolds were non-cytotoxic and supported fibroblast viability; BGAE constructs showed greater cell coverage over 14 days when surface-seeded and more stable fluorescence intensity through 28 days when encapsulated. In a murine thermal burn model, BGAE-treated wounds demonstrated more advanced re-epithelialization and more continuous epidermal coverage at day 14 compared to controls. These findings indicate that dual-stage crosslinking enables WJ-ECM integration while preserving printability, offering a practical platform for bioactive skin tissue engineering applications.

## 1. Introduction

The skin serves as a vital protective barrier, maintaining homeostasis and shielding against environmental insults [1]. Severe skin injuries, including deep burns, pressure ulcers, and chronic wounds, compromise this protective function, leading to serious complications and diminished patient quality of life. Conventional treatments, such as autologous skin grafts and synthetic dressings, face several limitations, including donor site morbidity, risk of immune rejection, and suboptimal regenerative outcomes [2,3]. These challenges have driven advances in tissue engineering, which aims to develop skin substitutes that closely replicate both the structure and biological functions of native skin [4]. Three-dimensional (3D) biological scaffolds (bio-scaffolds) have emerged as platforms for facilitating cell adhesion, proliferation, and differentiation, ultimately promoting tissue regeneration [5].

Among various scaffold fabrication techniques, 3D bioprinting has emerged as a promising approach due to its ability to construct precise architectures, incorporate living cells, and maintain biocompatibility [3,6,7]. Central to this approach is the development of suitable bioinks that balance printability, mechanical stability, and bioactivity to stimulate tissue repair [3,7,8]. Natural polymers such as gelatin and alginate are widely used in bioink formulations due to their biocompatibility and gelation properties. Gelatin, a collagen derivative, promotes cell adhesion and proliferation. Alginate, a polysaccharide extracted from brown seaweed, provides mechanical strength upon crosslinking with divalent cations, typically calcium ions [7,9]. Ionic crosslinking of alginate with Ca^2+^ proceeds through cooperative binding of calcium ions to guluronate blocks, forming stable “egg-box” junctions that constitute a physically crosslinked network [10]. The mechanical properties and swelling behavior of the resulting hydrogel are directly governed by crosslink density, which depends on calcium concentration, exposure time, and the molecular weight distribution of the alginate chains [11]. In gelatin-alginate composite systems, the interpenetrating polymer network formed by physical gelation of gelatin and ionic crosslinking of alginate provides complementary structural contributions: gelatin imparts thermoresponsive behavior and cell-adhesive peptide sequences, while alginate governs network stiffness and degradation kinetics [7,9]. However, bioinks composed solely of gelatin and alginate lack the biochemical complexity of native ECM, providing limited cues for cell proliferation, differentiation, and angiogenesis. Additionally, achieving consistent extrusion behavior requires careful modulation of crosslinking conditions: insufficient crosslinking yields low-viscosity formulations with poor shape retention, whereas excessive ionic crosslinking may induce heterogeneous gelation and compromise cell viability. Balancing printability with biological functionality thus remains a central challenge in developing gelatin-alginate-based bioinks.

Supplementing bioinks with ECM components has emerged as a promising strategy to enhance their tissue regenerative potential. Among various ECM sources, Wharton’s jelly-derived ECM from the human umbilical cord stands out as an attractive candidate due to its primitive nature, rich bioactive content, and strong regenerative capabilities [12,13]. Wharton’s jelly is a specialized connective tissue in the umbilical cord that contains high levels of collagen, glycoproteins, glycosaminoglycans (GAGs), and growth factors—components known to promote cell adhesion, proliferation, and angiogenesis [12,14]. Compared to ECM derived from xenogeneic or adult tissues, WJ-ECM offers immunological advantages, with lower antigenicity that may enhance compatibility in regenerative applications [15]. Furthermore, umbilical cords represent an abundant and ethically accessible source of regenerative biomaterials, as they are routinely discarded following delivery [13].

Previous studies have demonstrated the regenerative potential of WJ-ECM across multiple applications. In vitro, WJ-ECM supported attachment and infiltration of mesenchymal stem cells [16] and fibroblast cells [17], while in vivo studies showed accelerated wound healing and tissue regeneration at epithelial barriers [17,18]. In biomaterial fabrication, WJ-ECM has been integrated with poly(ε-caprolactone) to create electrospun 3D nanofiber scaffolds exhibiting favorable cell adherence and biocompatibility [19], and combined with gelatin methacrylate (GelMA) for 3D-bioprinted cartilage-mimicking substitutes for articular cartilage defect repair [20]. Despite these advances, the incorporation of WJ-ECM into extrusion-based gelatin-alginate bioinks for acellular skin scaffold fabrication has not been reported. Specifically, no prior study has examined a staged ionic crosslinking strategy that simultaneously preserves extrusion performance and structural fidelity while integrating WJ-ECM at biologically relevant concentrations.

In this study, we developed a dual-stage CaCl_2_ crosslinking strategy to enable the controlled incorporation of WJ-ECM into a gelatin-alginate bioink. A mild pre-printing CaCl_2_ concentration was employed to modulate viscosity and stabilize extrusion behavior, followed by secondary post-printing crosslinking to reinforce scaffold architecture. We hypothesized that WJ-ECM supplementation would enhance cellular compatibility and promote favorable histological regeneration in a murine burn wound model while maintaining extrusion performance and structural integrity comparable to baseline formulations. To test this hypothesis, we conducted physicochemical characterization, in vitro cytocompatibility assessment, and in vivo therapeutic evaluation using a murine thermal burn model.

## 2. Materials and Methods

### 2.1. Materials

Wharton’s jelly decellularized ECM from a human umbilical cord was provided by the Laboratory of Tissue Engineering and Biomedical Materials, University of Science, VNU-HCM (Ho Chi Minh City, Vietnam). L929 mouse fibroblasts were obtained from ATCC (Manassas, VA, USA). Human fibroblasts (hFs) were sourced from the same laboratory. Type B gelatin (bovine skin), sodium alginate (medium viscosity), pepsin, MTT reagent, DMSO, HCl (0.01 N), DMEM/F12, FBS, penicillin-streptomycin, and crystal violet were purchased from Sigma-Aldrich (St. Louis, MO, USA). CaCl_2_ and NaOH (0.1 N) were obtained from Merck (Darmstadt, Germany). PBS (10×) and LIVE/DEAD™ Viability/Cytotoxicity Kit were obtained from Thermo Fisher Scientific (Waltham, MA, USA).

### 2.2. Preparation of Decellularized WJ-ECM Solution

Wharton’s jelly ECM was decellularized via seven freeze (−86 °C, 60 min)/thaw (37 °C, 30 min) cycles and provided by the Laboratory of Tissue Engineering and Biomedical Materials, University of Science, VNU-HCM. Following our previous protocol [21], with modifications, lyophilized dECM was mechanically ground (Bead Ruptor 24, Omni International, Kennesaw, GA, USA) and solubilized in 0.01 N HCl containing pepsin (1 mg/mL) with stirring at 500 rpm for 72 h. The solution was neutralized to pH 7.0–7.4 on ice using 0.1 N NaOH and 10× PBS, yielding a 10 mg/mL pre-gel ECM solution. All procedures were performed under sterile conditions (Class II biosafety cabinet, ESCO, Singapore).

### 2.3. Bioink Preparation

#### 2.3.1. Preparation of Gelatin-Alginate Bioink (BGA)

Following our previous protocol [22], type B gelatin (10% *w*/*v*) and sodium alginate (10% *w*/*v*) were prepared in ddH_2_O and autoclaved (Hirayama HVE-50, Saitama, Japan). Equal volumes were mixed while warm, then sterile 70 mM CaCl_2_ was added at 1:1:0.5 (*v*/*v*/*v*) ratio (gelatin:alginate:CaCl_2_) with continuous stirring (500 rpm, 15 min), yielding 4% gelatin, 4% alginate, and 14 mM CaCl_2_. The bioink was transferred to 3 mL syringes, centrifuged (3000 rpm, 5 min) to remove air bubbles, and stored at 4 °C for up to one week.

#### 2.3.2. Preparation of WJ-ECM-Supplemented Bioink (BGAE)

BGAE followed the BGA procedure with pre-adjusted volumes to accommodate ECM. After cooling to 37 °C, solubilized ECM (1 mL, 10 mg/mL) was added under mild stirring (100 rpm, 5 min), followed by incorporation of sterile 70 mM CaCl_2_ at the same ratio as BGA. Final composition: 4% gelatin, 4% alginate, 14 mM CaCl_2_, and 1 mg/mL WJ-ECM. ECM concentration was based on prior evidence that 0.5–1 mg/mL maintains printability while supporting cell performance [23].

**Figure 1:**
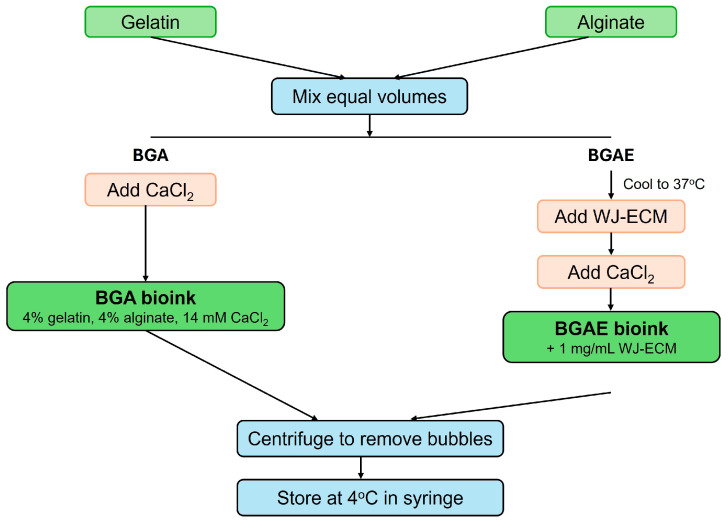
Schematic diagram illustrating the preparation procedures for BGA and BGAE bioinks.

The preparation procedures for both bioinks are summarized in Figure 1.

### 2.4. Bioink Characterization

#### 2.4.1. Viscosity Assessment

Apparent viscosity was measured using a Brookfield DV-III viscometer (10 mL samples, 25 ± 2 °C, spindle speed 0.07 s^−1^). Viscosity values were recorded across the applied shear stress range and reported as apparent viscosity (kcP).

#### 2.4.2. Inverted-Vial Test

BGA and BGAE bioinks (2.0 mL) were transferred to vials, rested 30 min at room temperature, then inverted 180°. Macroscopic flow was assessed at 5, 15, 30, and 60 min.

#### 2.4.3. Printability Evaluation

**Figure 2:**
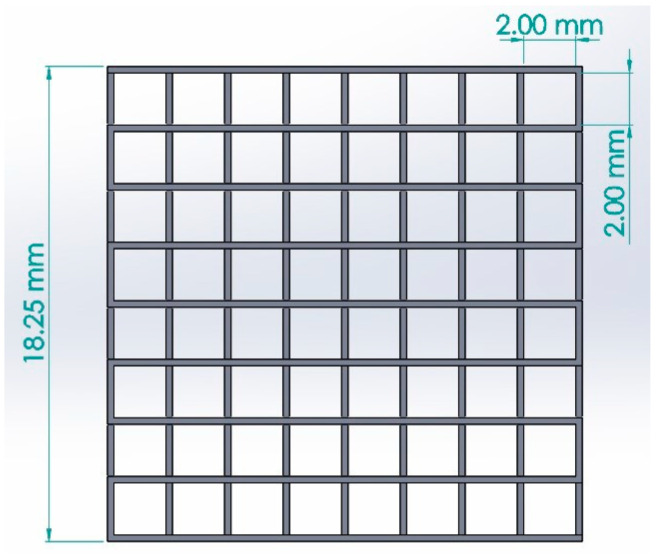
Computer-aided design (CAD) model for pore printability.

Printability was evaluated under extrusion conditions (25 G nozzle, 0.3 µL/mm, 5 mm/s, 25 ± 2 °C) for BGA, BGAE (with CaCl_2_), and control (gelatin-alginate without CaCl_2_). Filament formation was measured as unsupported filament length by air extrusion. Pore printability was assessed on square-grid prints (Figure 2) and quantified by printability index (Pr). Filament collapse was tested across 1–6 mm gaps.

### 2.5. 3D Printing and Crosslinking of Scaffolds

**Figure 3:**
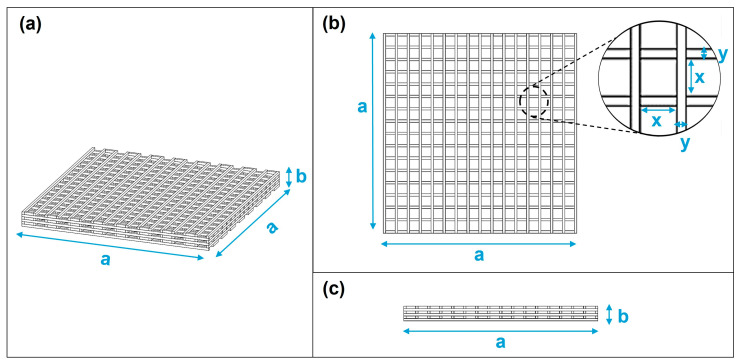
CAD model of the scaffolds. (**a**) Isometric view. (**b**) Top view. (**c**) Side view. a: width = length. b: height. x: designed pore size. y: filament size.

Three scaffold models with square-grid patterns were designed using CAD software. Models 1–3 were designed with a constant height (b = 1.5 mm) and theoretical filament diameter (y = 0.25 mm) but differed in their overall side length (a) and pore size (x), as specified in Table 1 and Figure 3. BGA and BGAE bioinks were printed using a BioX 3D printer (Cellink, Gothenburg, Sweden) with a 25G nozzle (0.25 mm inner diameter) at 0.3 μL/mm and 5 mm/s. Scaffolds were post-crosslinked in 0.5 M CaCl_2_ (30 min), then rinsed with 1× PBS and distilled water. The printed scaffolds derived from BGA and BGAE bioinks are hereafter referred to as SGA and SGAE, respectively.

### 2.6. Physicochemical Characterization of Scaffolds

#### 2.6.1. Fourier Transform Infrared Spectroscopy (FTIR)

FTIR spectra were recorded to confirm chemical composition and functional groups. Spectra were collected over 4000–400 cm^−1^ in ATR mode and by KBr pellet method using a MIR/NIR Frontier spectrometer (PerkinElmer, Shelton, CT, USA).

#### 2.6.2. In Vitro Degradability

Scaffolds were dried, weighed (*W*0), placed in complete medium (DMEM/F12, 10% FBS, 1% penicillin-streptomycin), and incubated at 37 °C, 5% CO_2_. On days 1, 4, 7, 14, 21, and 28, samples were retrieved, rinsed, surface-dried with filter paper, and weighed (*Wt*). Degradations were calculated as [24]:

(1) $$\%Degradation=\;\frac{W0-Wt}{W0}\;\times 100\%,$$

From the degradation percentage, the remaining mass percentage of the scaffold could be determined as:

%*Remaining mass* = 100% − %*Degradation*, (2)

### 2.7. In Vitro Biological Evaluation

#### 2.7.1. Cytotoxicity Assays

Cytotoxicity was evaluated per ISO 10993-5 [25] using two methods.

Direct contact method: L929 cells (5 × 10^4^ cells/well, 6-well plates) were cultured for 24 h. Scaffolds were placed centrally (~1/10 surface area) for 24 h, removed, and cells were stained with 0.5% crystal violet and graded 0–4 based on morphology.

Extract assay method: Scaffold extracts were prepared in complete medium (3 cm^2^/mL, 24 h, 37 °C) [23]. L929 cells (10^4^ cells/well, 96-well plates) were exposed to extracts, positive control (20% DMSO), or negative control (complete medium) for 24 h, then incubated with MTT (0.5 mg/mL, 4 h). Formazan was dissolved in DMSO:ethanol (1:1, *v*/*v*) and absorbance was measured at 570 nm (EZ read 400, Biochrom, Holliston, MA, USA). Relative growth rate (*RGR*%) was calculated:

(3) $$\%RGR=\;\frac{OD\;sample-blank}{OD\;negative\;control-blank}\;\times 100,$$

#### 2.7.2. Cell Viability and Attachment on Scaffolds

Cell seeding on surface: Scaffolds were placed in 24-well plates. Human fibroblasts (500 μL, 10^5^ cells/mL) were seeded and incubated at 37 °C, 5% CO_2_. Viability was assessed at days 1–14 by calcein AM staining.

Cell encapsulation: hFs were mixed into the bioink at a density of 10^6^ cells/mL to create a cell-laden bioink, which was then printed to form scaffolds containing embedded cells. The printed scaffolds were crosslinked with 0.5 M CaCl_2_ for 3 min, washed, and cultured at 37 °C, 5% CO_2_ for up to 28 days. At designated time points (days 1, 4, 7, 11, 14, 18, 21, and 28), scaffolds were retrieved and assessed by calcein AM staining.

Calcein AM staining: Samples were washed with PBS, stained with 2 µM calcein AM, and incubated (30 min, 37 °C, 5% CO_2_) for 30 min. Viable cells were visualized by fluorescent microscopy (Olympus, Tokyo, Japan).

### 2.8. In Vivo Wound Healing Evaluation

All animal procedures were approved by the Institutional Animal Care and Use Committee of the University of Science in Ho Chi Minh City, Vietnam (Approval No.: 1692/GXN-KHTN-ACUCUS). A total of 12 male Mus musculus mice with a body weight of 28–32 g at the start of the experiment were used. The animals were housed under controlled conditions (23 ± 3 °C, 12:12 h light:dark cycle) with ad libitum access to food and water. The cages were cleaned every two days and enriched with bedding material, gnawing items, and simple shelters to support natural behaviors.

Thermal burn injuries were induced under anesthesia by intramuscular injection of ketamine (60 mg/kg) and xylazine (8 mg/kg). After dorsal hair removal and skin disinfection, two circular burns were created on the dorsum of each mouse using a cylindrical metal rod (1 cm diameter) preheated to 100 ± 1 °C and applied for 15 s [26]. Twenty-four hours after injury (day 1), animals were randomly assigned to treatment groups. Burn wounds were treated with either gelatin-alginate scaffold (SGA) or WJ-ECM-supplemented scaffold (SGAE). At designated time points (days 7 and 14 post-injury), three mice per group were euthanized for tissue collection. For baseline histological characterization, three mice were sacrificed at day 1 prior to treatment. The harvested tissues were fixed in 10% neutral-buffered formalin, paraffin-embedded, sectioned (3–5 µm), and stained with hematoxylin and eosin.

### 2.9. Statistical Analysis

All experiments were performed with at least three independent replicates. Data are presented as mean ± standard deviation (SD). Statistical comparisons were conducted using unpaired two-tailed Student’s *t*-test for two groups, one-way ANOVA followed by Tukey’s multiple comparisons test for pore size analysis and cytotoxicity assessment, or two-way ANOVA followed by Tukey’s multiple comparisons test for degradation analysis. A value of *p* < 0.05 was considered statistically significant. All analyses were performed using GraphPad Prism 9 (GraphPad Software, Boston, MA, USA).

## 3. Results

### 3.1. Viscosity and Printability of WJ-ECM-Supplemented Bioinks

**Figure 4:**
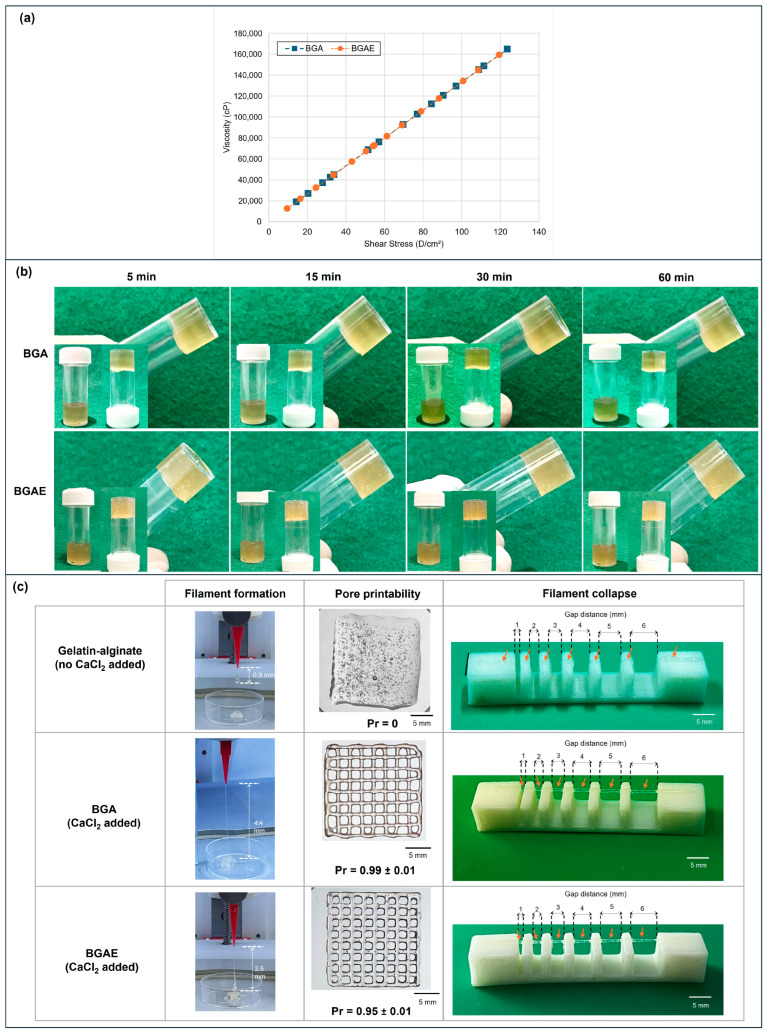
Viscosity and printability of BGA and BGAE bioinks. (**a**) Apparent viscosity of BGA and BGAE under varying shear stress. (**b**) Inverted-vial images at 5, 15, 30, and 60 min at room temperature. (**c**) Printability evaluation including air-extruded filaments, pore printability, and filament-collapse test. Orange arrows indicate printed filaments.

Both BGA and BGAE bioinks exhibited apparent viscosity values ranging from 20 to 180 kcP across the measured shear stress range (Figure 4a). BGA showed slightly higher apparent viscosity than BGAE across most measured points; however, the overall profiles remained comparable, indicating that incorporation of 1 mg/mL WJ-ECM did not substantially alter bulk flow resistance under the applied conditions.

To assess structural stability under gravitational load, an inverted-vial assay was performed. Both formulations maintained their shape without visible flow for up to 60 min at room temperature (Figure 4b), confirming sufficient pre-crosslinked network integrity to resist gravitational deformation.

Extrusion testing demonstrated that CaCl_2_-containing bioinks produced continuous filaments and well-defined grid architectures (Figure 4c). Printability indices were 0.99 ± 0.01 for BGA and 0.95 ± 0.01 for BGAE, both approaching the ideal value of 1.00 and confirming stable strand formation. In contrast, the CaCl_2_-free control exhibited filament disruption and structural collapse, demonstrating that pre-crosslinking is a prerequisite for scaffold fabrication and supporting the rationale for the two-step crosslinking strategy adopted in this study. Together, these findings indicate that mild pre-crosslinking conferred adequate viscosity and shape retention to support reproducible extrusion-based printing for both formulations.

### 3.2. Structural Optimization and Physicochemical Characterization of Printed Scaffolds

#### 3.2.1. Structural Optimization of Printed Scaffolds

**Figure 5:**
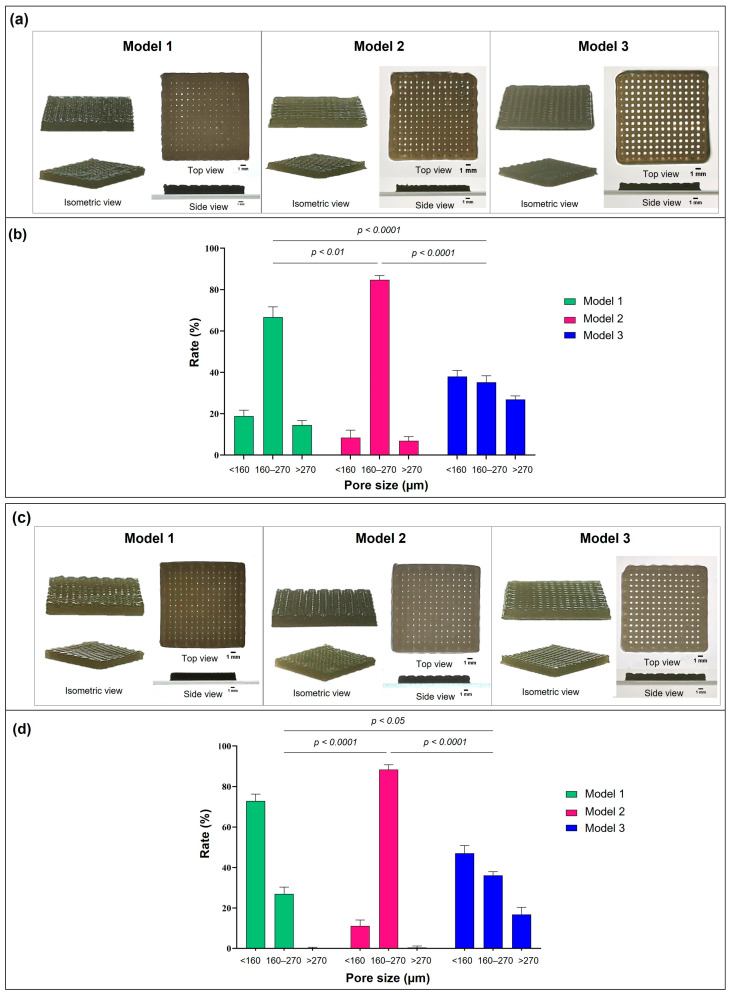
Structural analysis of SGA and SGAE scaffolds. (**a**) Morphological observation of SGAs. (**b**) Pore size distribution ratio of SGAs. (**c**) Morphological observation of SGAE scaffolds. (**d**) Pore size distribution ratio of SGAE scaffolds.

Three scaffold designs with nominal pore sizes of 0.98, 0.99, and 1.00 mm were printed to assess the correspondence between designed and realized pore architecture after printing and crosslinking (Figure 5). Although overall scaffold thicknesses were comparable among models in both SGA and SGAE groups, notable differences were observed in pore preservation within the predefined target range (160–270 µm). Measured pore dimensions were consistently larger than nominal design values across all models, indicating dimensional deviation following extrusion and post-printing stabilization. Overall scaffold thicknesses remained comparable among models in both SGA and SGAE groups.

Clear differences were observed in pore distribution within the predefined target range (160–270 µm). For SGA scaffolds, Model 2 achieved the highest proportion of target-range pores (84.72 ± 2.09%), compared with 66.66 ± 5.00% for Model 1 and 35.19 ± 3.14% for Model 3. Statistical analysis revealed no significant difference between Models 1 and 2 (*p* > 0.05), whereas Model 3 showed significantly lower proportions compared with both Model 1 (*p* < 0.05) and Model 2 (*p* < 0.01), indicating that the largest nominal pore size resulted in the greatest architectural deviation from the target range.

For SGAE scaffolds, Model 2 similarly achieved the highest proportion (88.39 ± 2.35%), compared with 26.96 ± 3.38% for Model 1 and 36.12 ± 3.51% for Model 3. Model 2 showed significantly higher target-range proportions than both Model 1 (*p* < 0.01) and Model 3 (*p* < 0.001), while no significant difference was observed between Models 1 and 3 (*p* > 0.05).

Although the difference between Models 1 and 2 did not reach statistical significance in SGA scaffolds, Model 2 was selected based on its numerically superior pore distribution and consistent performance across both SGA and SGAE formulations and was therefore used for all subsequent physicochemical and biological evaluations.

#### 3.2.2. FTIR Spectroscopy

**Figure 6:**
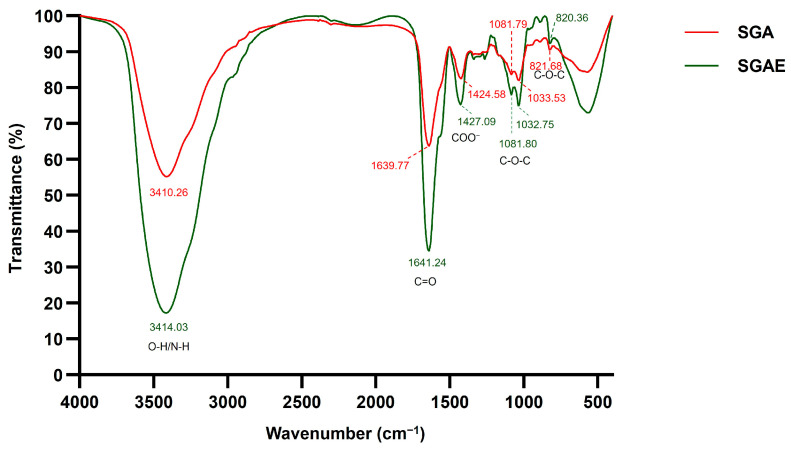
FTIR spectra of SGA and SGAE scaffolds.

FTIR spectra of SGA and SGAE scaffolds exhibited characteristic absorption bands corresponding to gelatin, alginate, and ECM components (Figure 6). Major peaks were observed at 3410–3414 cm^−1^ (O-H/N-H stretching), 1640–1641 cm^−1^ (amide I), 1425–1427 cm^−1^ (symmetric COO^−^), 1082 and 1033 cm^−1^ (C-O-C stretching), and 820–822 cm^−1^ (mannuronic acid), consistent with previously reported profiles [27,28,29,30,31,32,33,34]. Peak positions were conserved between formulations, with shifts not exceeding 4 cm^−1^, indicating preservation of the primary gelatin-alginate backbone.

Compared with SGA, SGAE scaffolds displayed lower transmittance (%T) values at most characteristic peaks, corresponding to relatively higher absorbance intensities. Notably, the O-H/N-H peak decreased from 55.18% to 17.19%, and the amide I band from 63.73% to 34.58%. Smaller differences were observed for the symmetric COO^−^ and polysaccharide-associated peaks, while the mannuronic acid signal remained comparable between groups. These spectral differences are consistent with the presence of additional ECM-derived proteins and glycosaminoglycans in SGAE, without detectable alteration of the primary gelatin-alginate network structure.

#### 3.2.3. In Vitro Degradability

**Figure 7:**
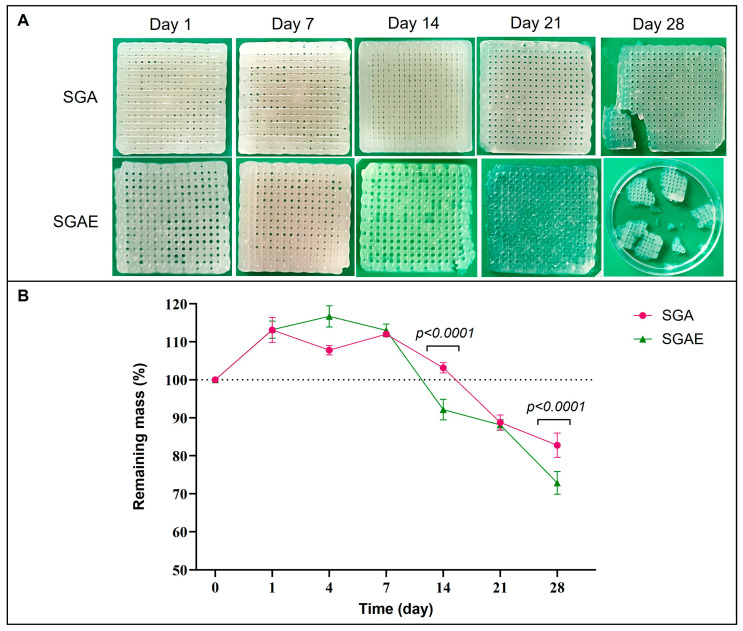
In vitro degradation of SGA and SGAE scaffolds over 28 days. (**A**) Macroscopic appearance at D1, D7, D14, D21, and D28. (**B**) Remaining mass (%) at different time points. *p* < 0.0001 (two-way ANOVA with Tukey’s post hoc test, *n* = 3). The dashed line indicates 100% remaining mass (D0).

Distinct degradation profiles were observed between SGA and SGAE scaffolds over 28 days (Figure 7). During the initial phase (D1–D7), both groups exhibited mass increases relative to dry weight, consistent with hydration-driven swelling. SGA reached a remaining mass of 113.11 ± 3.30% at D1 and stabilized at approximately 112% through D7, whereas SGAE peaked at 116.69 ± 2.79% at D4 before returning to 112.99 ± 1.68% at D7.

Following D14, progressive mass reduction was observed in both groups. SGA retained 103.15 ± 1.36% at D14, decreasing to 88.75 ± 1.98% at D21 and 82.76 ± 3.19% at D28. SGAE showed a more pronounced decline, reaching 92.14 ± 2.73% at D14, 88.09 ± 1.39% at D21, and 72.84 ± 3.00% at D28. Significant differences were observed between SGA and SGAE at D14 and D28 (Figure 7B, *p* < 0.0001), indicating that WJ-ECM incorporation resulted in accelerated scaffold degradation.

Macroscopic observations were consistent with quantitative measurements. SGA maintained structural integrity through D21 with minor fragmentation at D28, whereas SGAE exhibited earlier structural loosening from D14, followed by increased transparency, softening, pore deformation, and eventual fragmentation by D28.

### 3.3. In Vitro Biological Evaluation

#### 3.3.1. Cytotoxicity

**Figure 8:**
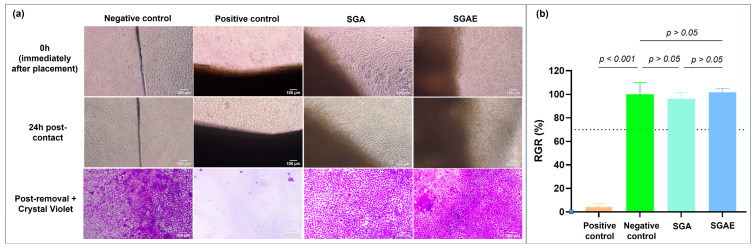
Cytotoxicity evaluation of SGA and SGAE scaffolds. (**a**) Crystal violet staining for direct contact assay. (**b**) Relative cell growth rate after exposure to scaffold extracts. The dashed line represents the 70% cell viability threshold according to ISO 10993-5.

Both direct contact and extract-based MTT assays were performed in accordance with ISO 10993-5 [25] to evaluate potential cytotoxic effects (Figure 8). In the direct contact assay, cells cultured in the presence of scaffold samples maintained normal morphology and uniform crystal violet staining comparable to the negative control, whereas the positive control exhibited characteristic cytotoxic responses, including cell rounding and detachment.

MTT analysis revealed relative growth rates (RGR) of 96.08 ± 5.13% for SGA and 101.64 ± 3.38% for SGAE. Neither value differed significantly from the negative control (*p* > 0.05 for both groups), and both exceeded the 70% viability threshold defined by ISO 10993-5 [25], confirming the absence of cytotoxicity under the tested conditions.

#### 3.3.2. Cell Viability and Attachment

**Figure 9:**
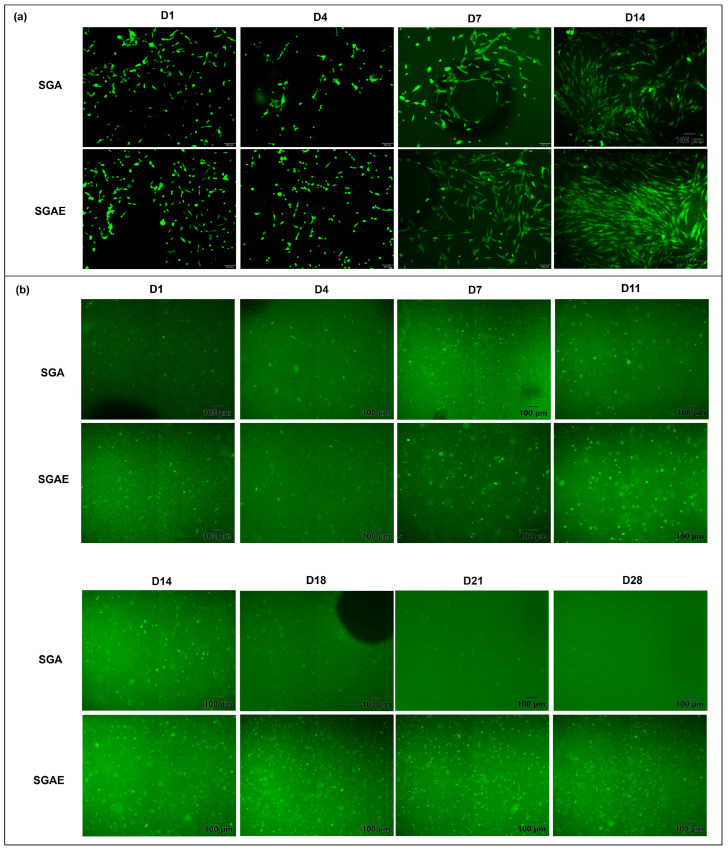
Cell viability and distribution on and within printed scaffolds. (**a**) Surface-seeded fibroblasts at D1, D4, D7, and D14. (**b**) Encapsulated fibroblasts within scaffolds at D1, D4, D7, D11, D14, D18, D21, and D28.

Fluorescence imaging of surface-seeded fibroblasts on printed scaffolds (Figure 9a) demonstrated successful cell attachment on both SGA and SGAE at D1, D4, D7, and D14. At early time points (D1–D4), cells were uniformly distributed and predominantly exhibited elongated morphology on both scaffold types. By D7 and D14, increased spreading and surface coverage were observed, indicating progressive cell-material interaction over time. At later stages, SGAE scaffolds showed visually greater surface coverage and cell density compared to SGA. Calcein-AM fluorescence staining of encapsulated fibroblasts (Figure 9b) demonstrated the presence of metabolically active cells within both scaffold types throughout the 28-day culture period. In SGAE constructs, fluorescence signals remained stable or gradually increased over time. In contrast, SGA constructs exhibited increasing fluorescence up to D14, followed by a progressive reduction at later time points (D18-D28), with signal intensity at D28 lower than that observed at D1. These observations suggest more sustained cellular metabolic activity in encapsulated SGAE constructs over extended culture.

### 3.4. In Vivo Wound Healing Evaluation

**Figure 10:**
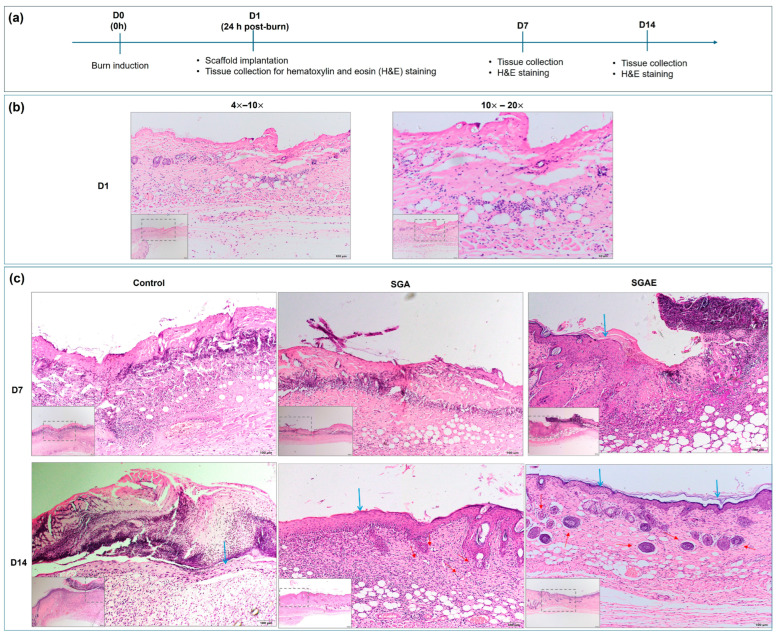
Wound healing progression in the burned mouse model with different scaffold treatments. (**a**) Experimental timeline. (**b**) Baseline histology at day 1 (24 h after burn; H&E; objective magnification: 4×–10× and 10×–20×). (**c**) Representative H&E sections at days 7 and 14 for the untreated control, SGA, and SGAE groups. Blue arrows: epidermis formation. Red arrows: neovascularization and dermal appendages. Grey dashed box indicates the region shown at higher magnification.

The experimental timeline is presented in Figure 10a, including burn induction (day 0), scaffold application (day 1), and tissue collection (days 7, 14). Histological examination on day 1 (Figure 10b) demonstrated complete epidermal loss and extensive dermal disruption, accompanied by a marked structural disorganization of the underlying adipose layer. These findings indicate a deep burn injury involving both epidermal and dermal compartments.

At day 7 (Figure 10c), early epidermal regeneration was primarily observed in the SGAE group (blue arrows), whereas other groups showed persistent epidermal disruption with eschar formation and limited, discontinuous neoepidermis. By day 14, control wounds displayed scab formation and irregular epidermal architecture without evident neovascular structures or dermal appendage formation. In contrast, SGA and SGAE groups exhibited more organized dermal-epidermal architecture with visible neovascular structures and identifiable dermal appendages (red arrows). The SGA group showed a comparatively thicker epidermal layer relative to adjacent normal tissue.

## 4. Discussion

This study developed a WJ-ECM-supplemented gelatin-alginate bioink using dual-step CaCl_2_ crosslinking for skin tissue engineering. The formulation maintained printability while exhibiting enhanced biological responses in vitro and in vivo, though the specific mechanisms and bioactive components underlying these improvements warrant further investigation.

The selection of 1 mg/mL WJ-ECM balanced biological augmentation with preserved extrusion performance. WJ-ECM provides type I and III collagen, glycosaminoglycans including hyaluronic acid, and proteoglycans—components that collectively provide cell-adhesive ligands (RGD motifs), regulate inflammation, and support angiogenesis [12,35,36]. Pepsin-based solubilization cleaves collagen telopeptides, generating smaller fragments that retain bioactive domains while minimizing viscosity increases [12,37]. This aligns with prior studies showing that 0.5–1.0 mg/mL ECM enhances cell adhesion without substantially altering gelatin-alginate rheology [23,38]. Our measurements showed that both BGA and BGAE exhibited viscosities of 20–180 kcP, within reported ranges suitable for extrusion-based bioprinting (30 cP–60 M cP) [39], with comparable profiles that translated to maintained printability and well-defined pore architectures. This preservation of flow properties is critical, as incorporation of bioactive molecules can modify bioink rheology in ways that hinder consistent extrusion [40].

The dual-step crosslinking strategy temporally separated deposition from mechanical reinforcement. Pre-print incorporation of 14 mM CaCl_2_ provided sufficient ionic crosslinking for shape-retaining filament deposition while preserving extrudability—creating a “printable gel state” viscous enough to maintain geometry yet fluid enough to extrude without clogging. Post-print immersion in 0.5 M CaCl_2_ (30 min for acellular scaffolds, 3 min for cell-laden constructs) substantially increased density and locked scaffold architecture. This two-environment approach enabled independent optimization of deposition behavior and mechanical stability—a flexibility that is challenging to achieve when crosslinking is performed in a single step. Subsequent washing removed excess calcium, preventing potential cytotoxicity [41].

The targeted pore size of 160–270 μm facilitates cellular infiltration, nutrient diffusion, and neovascularization [24,42,43,44], with pores in this range shown to support vascular formation within 14 days [45]. Our optimized design (Model 2) achieved > 84% of pores within this range for both SGA and SGAE, with 1.5–2.0 mm thickness approximating human dermal layer. The slight difference between SGA (84.72 ± 2.09%) and SGAE (88.39 ± 2.35%) may reflect subtle alterations in filament spreading, as ECM components can influence surface wetting [46,47,48]. However, both achieved pore architectures within the functional target, supporting our interpretation that 1 mg/mL WJ-ECM enriches biological functionality without disrupting physical structure.

FTIR analysis supported WJ-ECM incorporation while indicating preservation of the primary gelatin-alginate structure. Both SGA and SGAE exhibited characteristic absorption bands with minimal peak shifts (≤4 cm^−1^), indicating preserved fundamental structure. SGAE showed reduced %T (higher absorbance) at major bands, particularly in O-H/N-H and amide I regions, consistent with additional ECM-derived proteins and glycosaminoglycans. The mannuronic acid peak remained comparable between formulations, suggesting the alginate backbone was unaffected. These findings suggest successful WJ-ECM incorporation without detectable alteration of the primary gelatin-alginate network structure.

Both scaffolds demonstrated cytocompatibility. In surface-seeded constructs, SGAE exhibited greater cell coverage and more continuous distribution over 14 days. In encapsulated conditions, SGAE maintained more stable fluorescence intensity through 28 days, suggesting improved long-term cellular retention. These observations may be associated with WJ-ECM incorporation. The increased amide I absorbance is consistent with higher collagen-associated signals, which may contribute to integrin-binding motifs. Additionally, GAGs—particularly hyaluronic acid—modulate the pericellular environment by influencing hydration and protein adsorption [36]. The combined presence of collagen-derived adhesion ligands and GAG-mediated microenvironmental modulation likely creates a more biomimetic cellular niche in SGAE scaffolds.

In vivo evaluation in a murine burn model revealed that at day 14, both scaffolds showed improved tissue organization compared to controls, with SGAE exhibiting more advanced re-epithelialization. Histological analysis revealed more continuous epidermal coverage and better organization of basal and cornified layers in the SGAE group. Control wounds displayed persistent scab formation and irregular epidermal architecture with limited neovascular structures [26,49]. In contrast, scaffold-treated groups demonstrated more organized dermal-epidermal architecture with visible neovascular structures and identifiable dermal appendages. These findings suggest a more favorable early tissue response in wounds treated with WJ-ECM-supplemented scaffolds. However, wound contraction rate and epidermal thickness were not quantitatively assessed in the present study, which may limit precise differentiation between re-epithelialization and contraction-associated wound closure. Further morphometric analyses would help provide a more comprehensive evaluation of wound healing progression.

The observed swelling behavior during D1-D7 is consistent with the hydrophilic nature of both gelatin and alginate networks, which readily absorb water upon immersion in aqueous media [11,50]. It should be noted that swelling behavior in the present study was evaluated indirectly via remaining mass (%) under hydrated conditions, rather than by the conventional equilibrium swelling ratio derived from dry-to-wet weight measurements; therefore, direct numerical comparison with published swelling data is limited. Nevertheless, the qualitative swelling trend observed during D1–D7 is consistent with previous reports on alginate hydrogel systems. The slightly higher peak swelling observed in SGAE (116.69 ± 2.79% at D4 vs. 113.11 ± 3.30% at D1 for SGA) may reflect the hygroscopic nature of GAG components in WJ-ECM, particularly hyaluronic acid, which has strong water-binding and moisture-retention properties and is known to enhance water uptake in hydrogel matrices [51,52]. Importantly, both scaffolds returned to comparable remaining mass values by D7, suggesting that the initial hydration phase stabilized within the first week of incubation. The accelerated degradation of SGAE relative to SGA beyond D14 can be interpreted from the perspective of the polymer network architecture. WJ-ECM-derived macromolecules may reduce effective crosslink density by interfering with alginate ionic interactions [10,53,54]. ECM incorporation may also alter network organization and intermolecular interactions. A less densely interconnected matrix would therefore be more susceptible to structural destabilization and mass loss during prolonged incubation, consistent with the more pronounced degradation observed in SGAE at D21 and D28. This accelerated degradation profile may be advantageous in wound healing applications, where scaffold resorption concurrent with tissue remodeling is desirable; however, the optimal degradation rate relative to the rate of dermal regeneration warrants systematic investigation in future studies [24]. Gelatin and alginate are widely regarded as biocompatible materials with a relatively low toxicity, and their degradation products are generally considered acceptable for biomaterial applications. However, direct chemical characterization of degradation by-products was not performed in this study and should be addressed in future work.

The collective results illustrate coherent structure-function relationships across multiple scales. FTIR analysis supported the incorporation of WJ-ECM while indicating preservation of the primary gelatin-alginate network structure. This molecular enrichment was associated with enhanced cell adhesion, potentially mediated by ECM-derived integrin-binding motifs, and corresponded with improved re-epithelialization at the tissue level. Importantly, BGA and BGAE exhibited comparable viscosity, printability, and scaffold architecture, indicating that biological enhancement was achieved without measurable compromise in processability.

Several limitations warrant acknowledgment. First, although surface-seeded constructs were evaluated up to 14 days and encapsulated constructs up to 28 days, the study primarily focused on cell viability and distribution rather than quantitative assessment of long-term proliferation, matrix deposition, or functional tissue maturation. Second, while FTIR confirmed ECM integration, quantitative biochemical assays for specific bioactive components (collagen, GAGs, growth factors) would enable more precise structure-function correlations. Third, mechanical properties were not characterized; given that mechanical cues influence cell behavior, assessing whether mechanical differences contribute to observed cellular responses is warranted. Fourth, our 14-day in vivo endpoint captured early re-epithelialization but not complete dermal remodeling. Extended studies incorporating quantitative morphometric and comprehensive histological analyses would provide a more complete assessment of long-term tissue regeneration and scaffold performance. Finally, while 1 mg/mL WJ-ECM proved effective, systematic dose–response studies would identify optimal concentrations balancing bioactivity and printability.

This study establishes a practical strategy for incorporating bioactive ECM into printable bioinks, achieving a balance between printability and biological functionality often elusive in bioprinting. The incorporation of WJ-ECM through dual-step crosslinking was associated with improvements at cellular and tissue levels, highlighting the potential of rational biomaterial design strategies. While extended studies are needed to assess long-term tissue maturation, mechanical properties, and complete dermal remodeling, this platform provides a foundation for further development toward clinically relevant bioprinted skin constructs.

## 5. Conclusions

This study demonstrated a dual-stage CaCl_2_ crosslinking strategy that enables the incorporation of WJ-ECM into a gelatin-alginate bioink while preserving extrusion performance and scaffold architecture. Pre-crosslinking with 14 mM CaCl_2_ facilitated reproducible filament deposition, and post-print stabilization in 0.5 M CaCl_2_ reinforced structural integrity, yielding scaffolds with printability indices exceeding 0.95 and over 84% of pores within the target range (160–270 µm). FTIR analysis confirmed WJ-ECM integration without disruption of the primary gelatin-alginate network. Biological evaluations demonstrated cytocompatibility for both formulations, with WJ-ECM supplementation associated with greater surface cell coverage, more stable long-term fluorescence in encapsulated constructs, and more advanced re-epithelialization in a murine thermal burn model at day 14. These findings indicate that the rational incorporation of bioactive ECM into printable bioinks can enhance biological functionality without compromising processability, offering a practical platform for further development of bioprinted skin tissue engineering constructs.

## Figures and Tables

**Table 1:** Geometrical parameters of the designed scaffold models.

Model	a (mm)	b (mm)	x (mm)	y (mm)
1	19.93	1.5	0.98	0.25
2	18.85	1.5	0.99	0.25
3	20.25	1.5	1.00	0.25

a: width = length; b: height; x: designed pore size; y: filament size.

## Data Availability

The original contributions presented in this study are included in the article. Further inquiries can be directed to the corresponding author.

## Abbreviations

The following abbreviations are used in this manuscript:

CAD	Computer-aided design
BGA	Gelatin-alginate bioink
BGAE	WJ-ECM-supplemented bioink
ECM	Extracellular matrix
SGA	gelatin-alginate scaffold
SGAE	WJ-ECM-supplemented scaffold
WJ-ECM	Wharton’s jelly-derived ECM
